# The Complex Molecular Picture of Gut and Oral Microbiota–Brain-Depression System: What We Know and What We Need to Know

**DOI:** 10.3389/fpsyt.2021.722335

**Published:** 2021-11-02

**Authors:** Catia Scassellati, Moira Marizzoni, Nadia Cattane, Nicola Lopizzo, Elisa Mombelli, Marco Andrea Riva, Annamaria Cattaneo

**Affiliations:** ^1^Biological Psychiatry Unit, Istituto di Recupero e Cura a Carattere Scientifico (IRCCS) Istituto Centro San Giovanni di Dio Fatebenefratelli, Brescia, Italy; ^2^Laboratory of Alzheimer's Neuroimaging and Epidemiology, Istituto di Recupero e Cura a Carattere Scientifico (IRCCS) Istituto Centro San Giovanni di Dio Fatebenefratelli, Brescia, Italy; ^3^Department of Pharmacological and Biomolecular Sciences, University of Milan, Milan, Italy

**Keywords:** major depressive disorder, gut phageome, gut microbiome, oral microbiome, brain, biological molecular processes

## Abstract

Major depressive disorder (MDD) is a complex mental disorder where the neurochemical, neuroendocrine, immune, and metabolic systems are impaired. The microbiota-gut-brain axis is a bidirectional network where the central and enteric nervous systems are linked through the same endocrine, immune, neural, and metabolic routes dysregulated in MDD. Thus, gut-brain axis abnormalities in MDD patients may, at least in part, account for the symptomatic features associated with MDD. Recent investigations have suggested that the oral microbiome also plays a key role in this complex molecular picture of relationships. As on one hand there is a lot of what we know and on the other hand little of what we still need to know, we structured this review focusing, in the first place, on putting all pieces of this complex puzzle together, underlying the endocrine, immune, oxidative stress, neural, microbial neurotransmitters, and metabolites molecular interactions and systems lying at the base of gut microbiota (GM)–brain-depression interphase. Then, we focused on promising but still under-explored areas of research strictly linked to the GM and potentially involved in MDD development: (i) the interconnection of GM with oral microbiome that can influence the neuroinflammation-related processes and (ii) gut phageome (bacteria-infecting viruses). As conclusions and future directions, we discussed potentiality but also pitfalls, roadblocks, and the gaps to be bridged in this exciting field of research. By the development of a broader knowledge of the biology associated with MDD, with the inclusion of the gut/oral microbiome, we can accelerate the growth toward a better global health based on precision medicine.

## Introduction

According to a recent report from the World Health Organization, the prevalence of major depressive disorder (MDD) is increased by more than 18% between years 2005 and 2015, and nowadays, globally, more than 300 million people suffer from this disorder (1). Despite the research in this field, we are still unable to state neither a common cause nor a unique deregulated molecular pathway. Although with the increasing number of available medical interventions, about 50% of patients do not respond to the first trial of antidepressant drugs and about 30% of patients do not respond to any medications (2). This supports the notion that the pathology is not only a brain but also a body disorder.

A plethora of scientific evidence has produced important results on the molecular bases of MDD, going far beyond the initial monoaminergic hypothesis starting in the 1950s. The different theories established successively focus on biological systems that are very different from each other, and none of the hypotheses proposed overcomes a major role in the development of MDD: from neurotrophic to subsequent neurodevelopmental, glutamatergic, GABAergic, inflammatory/immune, oxidative stress; kynurenine (Kyn) pathway; clock gene machinery; and opioid and endocrine hypotheses (3, 4). As MDD is a complex and multifactorial disorder, these several biological processes likely contribute all together to the development of the disease, interacting with mutual reinforcing effects. MDD is also influenced by environmental factors that probably cause disruptions of these biological systems in different ways from individual to individual, making complex the clinical and etiopathological picture of this disorder. If we conducted a rough research in PubMed using the search terms such as “Major Depressive Disorder” AND “biological mechanisms,” we found, in the last 2 years, 38 reviews on the topic [including an article in Russian (5)], indicating a continuous research on this devastating disease.

Recent investigations have suggested that the development of mood and depressive behavior could be also influenced by the impact of gut microbiome (GM) on the central nervous system (CNS) functions (6), adding it as a further actor implicated in MDD. Interestingly, some antidepressant drugs display antimicrobial effects (7) and more than 50% of intestinal pathologies, such as irritable bowel syndrome, are actually treated with antidepressants (8). Specifically, Lukic et al. (9) demonstrated that the antidepressants (i.e., serotonin and/or norepinephrine reuptake inhibitors) showed common effects on the composition of GM, suggesting the possibility that gut bacteria mediate their therapeutic effects. For example, the decreased abundance of *Ruminococcus flavefaciens* induced by antidepressants has been associated with reduced depressive behavior. Conversely, *R. flavefaciens* administration upregulated genes involved in mitochondrial oxidative phosphorylation and downregulated genes involved in neuronal plasticity in specific brain regions including the medial prefrontal cortices (9). This suggests a mechanism for microbial regulation of antidepressant treatment efficiency.

The GM is composed of 10^3^ microorganisms, such as bacteria, fungi, viruses, and bacteriophages (phageome), which inhabit the gut (10), with over 1,000 unique bacterial species containing up to 20 million unique genes. It represents a sort of *ex corpore* organ, which normally establishes a symbiotic relationship with its human host: indeed, while gut microorganisms receive nutrition and protection, in return, the human body gains metabolites and neuroactive compounds (11). Their presence, products, abundance, and variability strongly influence the human host's physiology and pathology. The evidence that the GM can affect behavior and cognition and, in turn, that the brain can indirectly influence GM composition, has significantly contributed to determine the well-accepted concept of gut-brain axis. During the last decade, several studies have linked alterations in the GM and this axis with a number of different pathologies, including brain disorders (12).

The recent introduction of animals grown in complete absence of microbes, i.e., germ-free (GF) mice, has allowed researchers to observe how and how much microbes can influence the development and the physiology of the brain (13). Sudo et al. (14) have been the first in demonstrating the gut-brain connection. They compared the hypothalamic–pituitary–adrenal axis (HPA) functionality in response to stress in GF mice, in mice with normal microbiota but without specific pathogens [specific pathogen free (SPF)], and in mice raised with a selected group of microorganisms (gnotobiotic). The authors observed that GF mice showed an exaggerated HPA stress response, in terms of increased plasma adrenocorticotropic hormone (ACTH) and corticosterone levels, along with reduced brain-derived neurotrophic factor (BDNF) expression in the cortex and hippocampus compared to SPF mice. Moreover, *Bifidobacterium infantis* monocolonization restored the normal activation of the axis, by decreasing both ACTH and corticosterone plasma levels, whereas *Escherichia coli* enhanced the already deregulated response, although the mutant strain of *E. coli* had no such effect (14).

It is currently assumed that we carry around more microbial cells daily than our own human cells. These microorganisms are compartmentalized in “niches” such as the oral cavity (oral microbiome). Although research has focused mainly on the role of GM in MDD, recent investigations have suggested that also oral microbiome might play a key role in MDD development.

Based on this background, we structured this review in two main sections. The first one focuses on GM, for which extensive work in animals and humans is available; the second on promising but under-explored areas of GM-related research (oral microbiome and gut phageoma). In particular, the first part includes the description of the role of GM in the development of MDD followed by an overview of the molecular mechanisms of the GM-brain axis found altered in depression. In addition, we report new horizons coming from GM-oral microbiome–brain-depression: how alterations in the oral microbiome composition can influence GM and how this can have a crucial role in mental health. Finally, we describe investigations regarding the gut phageome or bacteria-infecting viruses. All this has the aim to clarify our understanding of this interesting and continuous evolving topic (14).

### Impairment of the Gut Microbiome Composition in Major Depressive Disorder

#### Evidence From Preclinical and Clinical Studies

Animal models represent key tools to investigate the biological mechanisms associated with the development of psychiatric conditions. Well-established early life stress paradigms in rodents include prenatal stress (PNS), maternal separation (MS) models, and social isolation during adolescence. It is also possible to take advantage of surgical interventions [olfactory bulbectomy, cerebral infusion of the corticotropin releasing hormone (CRH)] that trigger the activation of the stress response axis.

Eleven studies summarized in (15) evidenced the involvement of GM in MDD tested in animal models.

Four-month-old male rats exposed to PNS showed an exaggerated HPA axis response to stress resulting in plasma corticosterone release and impairments in cognitive functions. PNS animals also reported alterations in the GM composition, as indicated by the increased abundance of *Oscillibacter, Anaerotruncus, Peptococcus*, and reduced abundance of *Lactobacillus* (16). Female offspring exposed to PNS showed an increased anxiety-like behavior and alterations in the cognition in adulthood together with long-term changes of the bacteria belonging to the Rikenellaceae and Bifidobacteriaceae families (17).

As PNS, the MS paradigm caused an aberrant HPA axis and immune system activation, and it has been strongly associated with a disturbed GM (18). Interestingly, the paradigm of MS induced the development of anxiety-like behavior in SPF mice and not in GF. The colonization of maternally separated GF mice with the microbiota from non-separated SPF mice promoted the development of an anxiety-like behavior. On the contrary, when non-separated GF mice were colonized with microbiota from MS or non-separated SPF mice, no changes in behavior were observed and the GM profile of the two groups was comparable. All these results suggested that the presence of both an adverse environment (MS) and gut microbes are needed for the development of a depressive or anxiety-like behavior (19). MS treatment also resulted in increased hyperactivity and hyperresponsiveness of rats to stress occurring later in life (20). Interestingly, MS rats displayed higher abundances in *Bacteroides, Clostridium subcluster XIVa*, and *Clostridium cluster XI* and lower abundances in *Bifidobacteria* and *Lactobacillales* as compared to non-separated rats. A stressful event later in life (i.e., CRH infusion) enhanced these differences between maternally separated and maternally non-separated rats suggesting that MS-related GM alterations could be exacerbated by other stressors encountered later in life (21, 22).

Long-term changes in the GM composition have been reported also in rats who underwent social isolation during adolescence (23). In this study, the GM alterations found in adolescence were associated with hippocampal inflammation during adulthood, suggesting that stressful experiences during this sensitive period could have a long-lasting impact on the development of different biological systems that could in turn influence the vulnerability to develop mental disorders later in life.

Park et al. (24) reported that bulbectomized mice displayed both anxiety-, depressive-like behavior and higher basal hypothalamic CRH compared with sham-operated rats. Stool metagenomic profiling evidenced a similarity of only the 49.1% between sham-operated and bulbectomized rats, suggesting a redistribution in the proportion of some bacteria phyla after the bulbectomy. Intracerebroventricular infusions of CRH for 28 days led to the same kinds of alterations in behavior, intestinal motility, and GM composition that were observed in bulbectomized mice. This links once more central alterations, such as the hyperproduction of CRH and the subsequent hyperactivation of the HPA axis with changes in intestinal microbiota.

More recently, animal models with a microbiome transplanted from patients with MDD resulted in impaired social behaviors, with an increase in susceptibility to depression, elevated inflammation, and defective neural functions (15).

Overall, these preclinical studies highlight the importance of the GM in MDD, which strengthens the implication of animal models of depression on microbiota investigations (15). More importantly, through replication in different animal models of depression, these findings provided strong evidence on the crucial role of a disturbed GM in inducing impaired behavior, increased susceptibility to mental disorders, and inflammation (15).

Concerning human studies, in line with literature (15, 25–30), recent systematic reviews and meta-analyses showed microbial ecological diversity (29, 31, 32) and taxonomical (29, 31–33) differences in patients with MDD.

The first studies that investigated this topic on human date back to the years 2014–2015–2016. One of those showed an under-representation of the order Bacteroidales and the family Lachnospiraceae and an over-representation of the genera *Oscillibacter* and *Alistipes* in MDD patients (34). Another study performed in MDD patients as compared to controls demonstrated that they showed higher abundance of Bacteroidetes and Proteobacteria and a reduction in Firmicutes and Actinobacteria. Furthermore, negative correlations between *Faecalibacterium, Clostridium XIVb*, the severity of depressive symptoms and serum BDNF levels were observed. Nevertheless, the authors did not take into account the effect of medications on the GM composition; thus, it remains unclear whether the observed changes in the GM composition were associated with the pathology or were mainly a consequence of the pharmacological treatments (35).

Another research found a significant reduction in both *Bifidobacteria* and *Lactobacilli* in MDD patients with no significant association between these two genera with medications and bacterial counts. One of the limitations of the study is that few patients and controls which were consuming fermented milk with probiotics were not excluded from the study; therefore, probiotics could have possibly influenced the GM composition in these subjects (36).

In the same year (2016), Kelly and collaborators reported that at phylum level, no significant differences were found in MDD patients as compared to controls. However, at genus level, MDD patients displayed higher abundance of *Eggerthella, Holdemania, Gelria, Turicibacter, Paraprevotella*, and *Anaerofilum* and lower abundance of *Dialister*. More importantly, fecal transplantation (FMT) from either depressed patients or healthy subjects to antibiotic-treated rats induced a depressive-like behavior but only in those rats who had been transplanted with fecal samples from depressed patients, suggesting a key role of the gut bacteria in transferring depressive symptoms (37).

Another work investigated the GM profile in a group of MDD patients and controls, showing a higher abundance in *Prevotella* and *Klebsiella* in MDD patients compared to controls, but not in *Streptococcus* and *Clostridium XI* (38).

To date, according to Loniewski et al. (30), there are 28 available studies on the topic: 16 observational (638 MDD patients in total) and 12 clinical (436 MDD patients in total) studies. Although sometimes different in the design and in the methodology used to measure the GM composition, these data suggest that some bacteria strains are recurrent in MDD patients, even though to date no specific microbiota profile has been unequivocally associated with the depressive phenotype (31).

Thanks to the enormous efforts made in recent years, the link between GM dysregulation and the risk of developing MDD as well as the persistence of depressive symptoms is now well-established. A growing interest is now aimed at exploring the bidirectional communication between the GM and the immune, endocrine, and neural systems implicated in the etiology and pathophysiology of MDD (39).

### Molecular Mechanisms and Biological Pathways Mediating the Interplay Among Gut Microbes, Brain Functioning, and Behavior in Major Depressive Disorder

To date, the mechanisms causing the effect of the microbiota-gut-brain axis on MDD remains still unclear. However, increasing evidence implies the important role played by endocrine, immune, neural, and metabolic pathways in the communication between gut microbes and the brain (40). In a PubMed research literature, we found 53 reviews in the last 2 years (2020–2021; keywords: “depression” AND “gut-microbiota”), indicating evolving interest and investigations regarding this important relationship.

#### Endocrine System

The HPA axis is the well-known biological system in mediating the stress response. After the first study performed by Sudo et al. (14), it is now clear that the development and function of the HPA axis is influenced by the compositional and functional status of GM. Stress affects the HPA axis activation provoking the production of CRH by the hypothalamus, which in turn determines the subsequent downstream production of ACTH by the pituitary and, ultimately, cortisol (corticosterone in rodents) by the adrenal glands. Exposure to stress also modifies GM, and similarly, GM can also influence the HPA axis: studies demonstrated that microbiome-mediated changes in glutamate, serotonin (5-HT), other neurotransmitters, and BDNF, all involved in MDD etiology, can influence the HPA axis (41).

After the HPA axis activation, the release of cortisol and catecholamines can affect: (1) gut permeability, (2) gut mobility, and (3) microbial composition. The increased permeability of the gut barrier and the decreased gut mobility define a leaky gut. A leaky gut lets bacterial endotoxins [i.e., lipopolysaccharide (LPS)] leak out of the gut lumen and enter blood circulation. Endotoxins initiate peripheral inflammation response. These events are strongly correlated with the evidence that raised LPS or corresponding immunoglobulin levels have been reported in MDD and that chronic stress is a significant risk factor for MDD.

#### Inflammation and Immune Response

Dysfunction in the CNS and endocrine system, inflammation, and the alterations in immune function have long been considered important pathogenic risk factors of MDD (42). The identification of the role of GM in the immune system and its bidirectional communication with the CNS has produced increasing awareness in the reciprocal interaction among inflammation and GM in MDD (40).

The intestinal bacteria stimulate the development of a competent immune system, and in turn, multiple immune cells cooperate to maintain immune tolerance within the intestine (43). A growing body of findings demonstrated that GM remains unstable during the neonatal life, creating a crucial “window of opportunity” for the development of the host immune system (44). This process generally takes about 4–6 weeks in mice pups and 2–3 years in babies, until the microbial community reaches a relatively stable status (45). Moreover, it has been shown that both a microbiota depletion by antibiotic treatment during the perinatal period and the complete absence of microbiota (GF mice) lead to a reduction in circulating and bone marrow neutrophils in early neonatal period, indicating that the presence of microbes is necessary for the maturation and the priming of the systemic immune system (46).

In the gastrointestinal tract, innate and adaptive immunity play critical roles as guardians that maintain pathogen-host homeostasis (47). Apart from this, GM can also directly influence brain microglia. Erny et al. (48) and Thion and Garel (49) showed that GF mice exhibited widespread defects in the maturation and function of microglia, and this resulted in deficient innate immune responses. Moreover, the authors demonstrated that a full repertoire of gut bacteria is necessary for a normal microglia development.

Different biological pathways can be activated when a peripheral inflammation, triggered by endotoxins, can propagate to the brain and initiate neuroinflammation: (1) peripheral cytokines send inflammatory signals to the brain *via* afferent nerves, (2) cytokines pass through permeable sections of the blood–brain barrier (BBB), and (3) activated immune cells migrate into the brain. It is important to underline that neuroinflammation may be especially insidious since it may last up to 40 times longer than the initial peripheral immune response (50).

There can be serious consequences once chronic inflammation develops in the brain. First, activated microglia and astrocyte release reactive nitrogen (RNS) and oxygen (ROS) species contributing to neural toxicity, since RNS and ROS damage brain epithelial cells and compromise the BBB (51). This inflammation can induce an increase in the production of interleukins (IL)-6 and IL-1β and in the inflammasome nucleotide binding and oligomerization domain-like receptor family pyrin domain containing 3 (NLRP3) in brain-resident cells. Second, other inflammatory cytokines [interferon-γ (IFN-γ), IL-6] can be responsible for the activation of the enzyme indoleamine-2,3-dioxygenase (IDO), increasing the metabolism of tryptophan (Trp) and, consequently, the release of neurotoxins (52). Third, neuroinflammation can inhibit the synthesis of the monoamine neurotransmitters 5-HT, dopamine (DA), and norepinephrine (NE) because pro-inflammatory cytokines damage and divert the activity of tetrahydrobiopterin, an essential enzyme co-factor of monoamine synthesis (53). In general, a chronic neuroinflammation stimulates a toxic environment inappropriate for proper brain activity and likely damaging for mental health.

#### Oxidative Stress System

The stimulation of the inflammatory pathway is, as reported above, characterized by a hyperproduction of ROS and RNS with a consequent damage of DNA, proteins, mitochondria and cell membranes. The detection in MDD patients of high levels of by-products of lipid peroxidation such as malondialdehyde and 4-hydroxynonenal (54) indicates the presence of an oxidative and nitrosative stress in MDD. On the other hand, GF mice presented a reduced antioxidant enzyme activity (55, 56). Moreover, an altered GM can stimulate the NADPH oxidase (57) and the nitric oxide synthesis (58), inducing oxidative stress. These altered mechanisms may lead to neuroinflammation and decreased neurogenesis and neuroplasticity (59).

In normal conditions, it is known that a class of ubiquitously expressed intracellular proteins, the heat shock proteins (HSP), safeguard the gut epithelial barrier from oxidative stress and inflammation. These proteins are chaperones and play a key role in the synthesis and folding of different proteins, contributing to their repair and stabilization. For these functions, evidence suggests that stressful conditions can increase the synthesis of the HSPs and their release (60), determining a stimulation of inflammatory response. It has been demonstrated that MDD patients showed high plasmatic concentration of extracellular HSP70, suggesting that HSPs could play a role in the occurrence of mood disorders (61). Interestingly, GM activity and diversity can influence the physiological epithelial HSP tone. Indeed, several *Bifidobacteria* and *Lactobacilli* are strong inducers of gut epithelial HSPs, promoting gut protection (62).

#### Neural Signaling

Autonomic nervous system dysfunction, with increased sympathetic and decreased parasympathetic (vagal) tones, is suggested to be an important contributing factor in the development of MDD (63).

The vagus nerve is the major parasympathetic nerve in the body and plays key roles in regulating several organ functions. It is linked to both the immune system and HPA axis, and it is a major mediator in the gut-brain axis. Vagal afferent nerve fibers are distributed throughout the intestinal wall, even though they are barred from direct contact with GM by the intestinal epithelial barrier (64). Thus, they respond to bacterial signals indirectly following exposure to bacterial neurometabolites such as neurotransmitters and short-chain fatty acids (SCFAs) and through interaction with gut enteroendocrine cells (65), which release hormones. Vagal nerves sense gut contractility information to CNS and interact with immune system with an anti-inflammatory effect (reduction of pro-inflammatory production and attenuation of the systemic inflammatory response) (66), sending afferent signals to the brain and activating an efferent response in cholinergic anti-inflammatory reflex which in turn activates HPA axis.

It has been demonstrated that an increased vagal activation occurs with probiotic supplementation and that vagotomy prevented the restorative effect of probiotics on anxiety (67, 68).

#### Neurotransmitters: Focus on Serotonin and the Aminoacid Tryptophan

One of the greatest risk factors for MDD is represented by the exhaustion of monoamine neurotransmitters. Indeed, most of the current antidepressants used for MDD treatment aims to increase the levels of monoamine neurotransmitters in the synapses. 5-HT, DA, and γ-aminobutyric acid (GABA) are the three main monoamine neurotransmitters. They play pivotal roles in maintaining homeostasis in the entire human body, as well as control the development and plasticity of neural circuits implicated in MDD (69). These neuroactive compounds can act locally on the enteric nervous system (ENS) but also directly on the brain either by crossing the BBB or communicating with vagal chemoreceptors (70). It is known that some bacteria strains are able to produce and/or interfere with the biosynthesis or the metabolism of these neurotransmitters (71). Indeed, *Lactobacillus* and *Bifidobacterium* secrete GABA; *Escherichia, Bacillus*, and *Saccharomyces* produce NE; *Candida, Streptococcus, Escherichia*, and *Enterococcus* produce 5-HT; *Bacillus* and *Serratia* can produce DA; *Lactobacillus* can secrete acetylcholine. This becomes important as all these neurotransmitters that can be regulated by the GM play an important role not only in MDD but also in antidepressant drugs mechanism (72).

5-HT is strictly connected to the gut: about 95% of the total 5-HT has origin within the gut, especially from the enterochromaffin cells, a subtype of epithelial cells of the digestive tract designated in modulating its motility (73). 5-HT is not merely a neurotransmitter involved in regulating mood, sleep and behavior, but it is also: (i) an endocrine hormone that exerts its effects on several organs, such as bone and liver; (ii) a paracrine factor, as it acts directly on enterochromaffin cells; (iii) a modulator of the immune system, as several of the 5-HT receptors have been found in lymphocytes, monocytes, macrophages, and dendritic cells; and (iv) a growth factor, as serotonergic neurons influence the development of other types of enteric neurons as well as the mechanisms of adult neurogenesis in the ENS.

5-HT synthesis depends on the availability of Trp, an essential amino acid, which must be supplied by the diet. Indeed, some bacteria are capable of metabolizing or producing the 5-HT precursor Trp and transform it into a compound (indole) typically used in microbial intercellular signaling, thus depleting the Trp availability to the host. Indole exposure can reinforce the mucosal barrier and mucin production by stimulating expression of indole target genes, thus increasing resistance to pathogen invasion. Moreover, indole exposure can suppress the production of pro-inflammatory cytokines and simultaneously increase the activation of anti-inflammatory ones, ameliorating inflammation and damage (74).

The relationship between gut, 5-HT, and brain is supported by several studies conducted in GF mice where the absence of the gut microbes was associated with higher Trp plasma levels, which can be normalized following colonization immediately post-weaning, and of higher 5-HT levels in the hippocampus, which conversely are not restored by an early colonization (75, 76). Therefore, GM may play a crucial role in Trp availability and metabolism to consequently impact central 5-HT concentrations (77). Importantly, GF mice display an inappropriate development of central serotonergic system (78).

The degradation route in the metabolism of Trp is represented by the Kyn pathway. The enzymes IDO and tryptophan-2,3-dioxygenase (TDO) catalyze the initial rate-limiting metabolic step of the Kyn pathway and lead to the production of Kyn. TDO is affected by stress-elevated glucocorticoids, whereas IDO levels are controlled by intestinal inflammation by pro-inflammatory stimuli (IFN-γ) (79). The final product is represented by neuroactive compounds as kynurenic acid (KYNA), anthranilic acid, and quinolinic acid. KYNA is thought to be a neuroprotective substance and acts as an N-methyl-d-aspartate NMDA agonist (80). Some studies showed decreased levels of Trp and an increased KYNA-to-Trp ratio in the plasma from MDD patients, as well as the ratio of KYNA to quinolinic acid (neurotoxic). Correlations have been also observed between these alterations in Trp metabolism and learning impairments and processing speeds in these patients, suggesting a potential link between the Kyn pathway and cognitive impairments in MDD (81).

#### Metabolites

Among the mechanisms mediating the effects of the GM on the brain, there is also the production of metabolites and compounds with neuroactive and immunomodulatory properties (82). Microbially derived metabolites include SCFAs, bile acids, choline and phenolic metabolites, indole derivatives, vitamins, polyamines, and lipids (83). The mechanisms through which these gut microbial metabolites can affect depressive behavior comprise: (1) direct stimulation of central receptors, (2) peripheral stimulation of neural, endocrine and immune mediators, and (3) epigenetic regulation of histone acetylation and DNA methylation (84).

Bacteria produce SCFAs as a byproduct of the fermentation of non-digestible carbohydrates. The resulting end products involve acetate, propionate, butyrate, and, to a lesser extent, iso-butyrate, valerate, and iso-valerate. The known salubrious properties of SCFAs can derive both from their lipophilic nature allowing them to easily reach the brain by crossing the BBB, where they interact with neurons, and from their strengthening properties of the intestinal barrier that is the major crossing point of molecules and nutrients from the bloodstream to the brain (82). They activate G-protein coupled receptors located on endocrine and immune cells, kidneys, blood vessels, and nerve cells.

The involvement of SCFAs in MDD etiopathogenetic mechanisms comes from different scientific evidence. In particular, SCFAs exhibit important anti-inflammatory and antidepressant (82) properties, and low SCFA levels, characteristic of a dysbiotic GM, are one of the causes of inflammation in MDD. In animal model experiments, it has been demonstrated that FMT from depressed patients to sterile rats resulted in increased fecal SCFAs levels compared to rats receiving microbiota from healthy subjects, and at the same time made them anxious (37).

Concerning findings related to sodium butyrate, the administration of this SCFA in an animal model of mania reestablished normal levels of activity and mitochondrial function in different brain regions such as the prefrontal cortex, hippocampus, striatum, and amygdala (85). Sodium butyrate also abrogated depressive-like and mania-like behavior in rats (86). Its antidepressant activity has been further demonstrated on a rat model of chronic mild stress, where its effects have been tested in relation to behavior, memory, and levels of neurotrophic factors (87). Sodium butyrate, aside from regulating the levels of neurotrophic factors, can inhibit histone deacetylation and prevent hippocampal microglia activation. Bacteria-derived butyrate has been also indicated to modulate the synthesis of DA, NE, and adrenaline (88).

Acetic, propionic, and caproic and valeric acids have been shown to partly contribute to the origin of symptoms of MDD (82). It has been reported that patients with MDD showed lower levels of acetate, an SCFA exerting protective activities against enteropathogenic infections and fortifying the gut barrier. This reduction was translated also into a decrease in butyrate acid. Similarly, lower levels of propionate, which has a key role in dampening the innate immune cell response to bacteria and keeping intestinal permeability in check (82), may contribute to dysbiosis and neuroinflammation observed in MDD. In the study by El-Ansary et al. (89) propionate administrated to animals produced alterations in phospholipid and acylcarnitine profiles. Moreover, propionate can modulate the secretion of 5-HT in the gastrointestinal system and cause a decline in brain 5-HT and DA levels (89). In addition, it has been observed that Polish women affected by MDD showed lower fecal levels of isocaproic acid as compared to controls (90). Valeric acid is a further SCFA found to be correlated with MDD (91), interfering with the release of neurotransmitters into the synaptic cleft. Interestingly, valeric acid presents a structural similarity to GABA, and thus, it can act as an inverse agonist of the adenosine A1 receptor in the brain, which is known for its role in the regulation of neurotransmitter release (91).

Finally, the GM is a supply of vitamins, including vitamins K and B, niacin, biotin, riboflavin, folate, and pyroxidine (83).

A less investigated metabolic pathway involved in the modulation of the GM composition and that influences mood is represented by the endocannabinoid (eCB) system (92). The GM influences both the eCB and the integrity of the intestinal barrier. Different studies support that a compromised integrity of the intestinal barrier is associated with MDD, causing the subsequent inflammation, and thus could be linked to an alteration of the eCB system, suggesting that probiotic intervention targeting this system in the gut can be used to improve host health.

### Oral Microbiome and Neuroinflammation in Major Depressive Disorder: Potential Link With Gut Microbiome

The oral microbiota is composed of many microorganisms stored in a complex environment that covers distinct and small microbial habitats, such as teeth, buccal mucosa, soft and hard palate, and tongue, which form a species-rich heterogeneous ecological system. About 50–100 billion bacteria have been found in the oral cavity, and 600 prevalent taxa, at the species level, with distinct subsets predominating different habitats. These species fit with 185 genera and 12 phyla, of which approximately 54% are officially named, 14% are unnamed (but cultivated), and 32% are known only as uncultivated phylotypes. Alterations in this ecosystem can influence systemic disorders: periodontal disease has been associated with Alzheimer's disease (AD), diabetes, metabolic syndrome, obesity, eating disorders, liver disease, cardiovascular disease, rheumatoid arthritis, and cancer (93). Interestingly, for common environmental (94–96) and genetic (97–101) risk factors, different findings link periodontal disease also to depression.

Recently, a study reported a first culture-independent investigation of the oral microbiome in depression and anxiety symptoms in adolescence. The results indicated that MDD and anxiety symptoms were associated with differential abundance of specific bacterial taxa, including *Spirochaetaceae, Actinomyces, Firmicutes, Treponema, Fusobacterium*, and *Leptotrichia* spp. (102), suggesting that oral microbiome composition can be associated with adolescent anxiety and depression symptoms. In addition, Wingfield et al. (103), detecting the structure and composition of the salivary microbiome in young MDD adults as compared with control subjects, evidenced that a total of 21 bacterial taxa were differentially abundant in the depressed cohort, including increased *Neisseria* spp. and *Prevotella nigrescens*, while 19 taxa had a decreased abundance. Both studies confirmed the involvement and a specific role of the oral microbiome in depression mechanisms in young adults.

Several studies supported the importance of oral–gut microbiome axis. Although the oral cavity and gut are continuous sections linked through the gastrointestinal tract, the oral and gut microbiome profiles are well-segregated, due to the presence of the oral–gut barrier, physical distance, and chemical impediments, such as gastric acid and bile. Nevertheless, the impairment of the oral–gut barrier can allow interorgan translocation and communication: the oral microbiota can translocate to the gut, and gut microbes can transmit to the oral cavity in inter- and intrapersonal manners, dependently on poor hygienic conditions. This bidirectional interaction can shape and/or reshape the microbial ecosystems in both habitats through either competition or cooperation, and this can influence the pathophysiological processes in the gastrointestinal tract (104).

What are the mechanisms that associate the oral and intestinal microbiota with the inflammation processes, hallmark of MDD? A local dysbiosis of oral and gut microbiota can impact not only on local tissues but also can affect distant organs, contributing in this way to the evidence that microbial elements may be associated with the development of neuroinflammation within the brain that in turn can reflect the MDD pathogenesis. Indeed, there are some components of oral microbiome for which different studies demonstrated their implications in neuroinflammation. *Porphyromonas gingivalis*, a gram-negative anaerobic bacterium, is part of the resident oral microbiome (105). A rise in its proportion as compared to other local microorganisms is associated with specific diseases such as periodontal disease and tissue destruction (106–108). Indeed, minor changes in its abundance within the biofilm can cause significant changes in the local ecosystem (109). *P. gingivalis* can modulate the host immune response by two systems: initially it can promote inflammation to increase nutrient availability and biofilm growth, and subsequently, it can facilitate bacterial resistance by destroying complement factors (110, 111). Interestingly, in a large national survey, it has been demonstrated that AD incidence as well as mortality risk were linked to a composite of *P. gingivalis* titers, and to *P. nigrescens* (112). This study thus suggested a significant association between periodontal pathogens and a disorder such as AD where the neuroinflammation processes play a crucial role in its etiology.

Spirochetes, helical-shaped motile bacteria, show a remarkable capability to penetrate tissues and spread infection (113). Treponema denticola is an important spirochete pathogen, as it can cause diseases such as periodontal disease and is associated with tissue destruction. Spirochetes are believed to activate Toll-like receptors (TLRs) on glial cells *via* CD14 and stimulate pro-inflammatory cytokine production, suggesting a potential involvement in neuroinflammation (114).

Fungi are another important component of the oral microbiome. *Candida albicans* are species found ubiquitously on oral surfaces and are a component of commensal biofilms. Due to imbalances caused by antibiotic usage or immunosuppressive conditions, they can overproliferate and cause local diseases (115). Interestingly, some studies demonstrated that fungal infections can also travel into the bloodstream and spread to distant tissues and organs. A recent work by Wu et al. (116) used a mouse model to generate *C. albicans* intravenous infections and noted the development of neuroinflammation around yeast cells. In the brain, *C. albicans* invasion also caused activation of transcription factor NF-κB and increased IL-1β, IL-6, and tumor necrosis factor (TNF-α) levels, suggesting that it can be responsible for the activation of local innate immune response. As a result of this neuroinflammation, infected mice showed mild memory impairment, which disappeared after antifungal treatment (116).

In the gut, specific bacterial strains can influence the neuroinflammation. For instance, *Akkermansia muciniphila*, a key regulator of inflammation in the gut (117), and *E. coli* (118) resulted in microglial activation that in turn is associated with an increase in TLR-2, TLR-4, TNF-α, and IL-1β levels.

**Figure 1:**
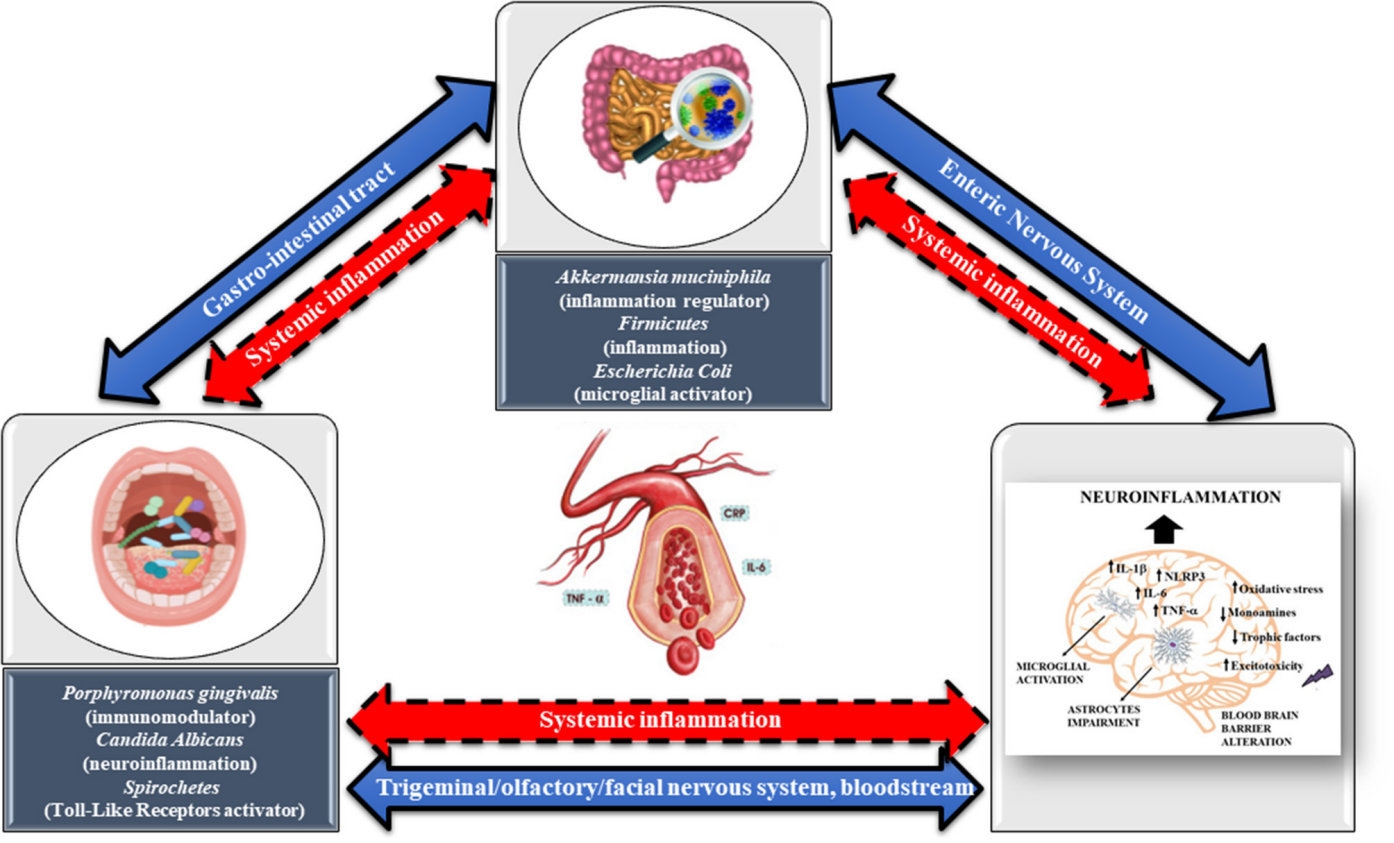
The direct mechanisms through which oral bacterial species and their products can impact the brain are represented by trigeminal/olfactory/facial nervous system and circulating blood (blue arrow, continuous line), whereas those indirect are through the involvement of GM dysbiosis and systemic inflammation (red arrow, dashed line, bidirectional). Likely, the direct mechanisms through with the GM and its products can affect the brain are the enteric nervous system (blue arrow, continuous line, bidirectional), whereas those indirect are through the mediation of systemic inflammation (red arrow, dashed line, bidirectional). Both direct and indirect effects coming from oral microbiota and GM can contribute to microglia-mediated neuroinflammation, resulting in related pathologies such as major depressive disorder. Neuroinflammation is mainly mediated by microglia, whereas perivascular myeloid cells and astrocytes play an auxiliary role. The activation of microglia promotes the release of several proinflammatory cytokines, including interleukin-IL-1β, IL-6, tumor necrosis factor (TNF)-α, chemokines, inflammasome NLRP3, and reactive oxidative species (ROS) (oxidative stress). Continuous and progressive inflammation can lead to the accumulation of many microglia and astrocytes at the site of inflammation, and the excessive release of pro-inflammatory cytokines will further exacerbate neuroinflammation and provoke synaptic toxicity and neuronal death. Reduction in monoamines, trophic factors, and excitotoxicity are also typical hallmarks of MDD.

Thus, the mechanisms through which oral bacterial species and their products can impact the brain can be either direct, such as through the trigeminal/olfactory/facial nervous system and bloodstream, or indirect, through the involvement of GM dysbiosis and systemic inflammation. Likely, the mechanisms through which the GM and its products can affect the brain can be either direct, through the ENS and the bloodstream, or indirect, through the mediation of systemic inflammation (119). Both direct and indirect effects can ultimately contribute to microglia-mediated neuroinflammation, resulting in related pathologies such as MDD (Figure 1). Neuroinflammation is a leading cause of nerve cell necrosis, and it is also a trigger mechanism for MDD (120). Neuroinflammation is mainly mediated by microglia, whereas perivascular myeloid cells and astrocytes play an auxiliary role. The activation of microglia promotes the release of several proinflammatory cytokines, including IL-1β, IL-6, TNF-α, chemokines, inflammasome NLRP3, and ROS (Figure 1). Continuous and progressive inflammation can lead to the accumulation of many microglia and astrocytes at the site of inflammation, and the excessive release of pro-inflammatory cytokines will further exacerbate neuroinflammation and provoke synaptic toxicity and neuronal death.

In the last section, we will focus on new and less explored topics that involve the investigations regarding the gut phageome.

## Gut Phageome

In the human gut, bacteria and their viruses form the most abundant biological entities (121). The virome includes both bacteriophages and eukaryotic viruses; however, phages (phageome) are significantly more abundant and drive microbiome composition, modulating its homeostasis (122).

The bacteriophage community in the human gut is a mixture of three classes: a set of core bacteriophages shared among more than one-half of all people, a common set of bacteriophages found in 20–50% of individuals, and a set of bacteriophages that are either rarely shared or unique to a person (123). To date, the existence of a core phageome is under debate, and the recent remarkable progresses in genome-based phage taxonomy (124, 125) may enable to better define the most common phage “types” in the human microbiota in the near future.

Healthy individuals tend to conserve the same phages over time (tested over 1 year), especially the most abundant ones (126). Persistent phages seem also to be more commonly shared than others (127). A common assumption is that newborns are born sterile. Rapid colonization of their newborn gut in the first days of life was reported by a dynamic assembly of bacteriophages (128). Progressive maturation of the infant's GM determines a reduction of viral abundance and diversity, along with an increase in abundance and diversity of the bacterial component.

The phages can be grouped by their lifecycle as either lytic or temperate. Temperate phages constitute from 20 to 50% of free phages in the human gut (126) and they can establish a long-term association with its host (lysogen). This phase is called lysogeny, where the mostly quiescent phage genome (prophage) is replicated by the bacterial machinery. Many prophages are highly stable; however, any environmental stressors or stochastic fluctuations can trigger their induction (resumption of the lytic cycle), eventually culminating in the destruction of their host cell.

The identity of the hosts targeted by the phages is still a research field to be explored. In 2016, Edwards et al. (129) discussed the comparison among different methodologies for host prediction. By analyzing 820 phages with annotated hosts, the authors found that sequence homology approaches are the most effective at identifying known phage–host pairs. Compositional and abundance-based methods contain significant signal for phage–host classification, in this way there is the opportunity for analyzing the unknowns in viral metagenomes. CRISPR spacers is another method widely used in different investigations to predict hosts [reviews in (126, 130)]. It leads to greatly confident predictions; however, on the other hand, it is restricted to hosts encoding CRISPR–Cas systems and where phage infection is relatively recent. Between 4 and 13% of phages could be assigned to a host in this way. A further program developed is WiSH that bases its predictions on the similarity of the phage genome to that of its hosts. It uses a probabilistic approach that compares the composition in subsequences of nine nucleotides, or 9-mers, in phage and bacterial genomes, reaching good prediction even for short 3-kb-long phage contigs. This is not performed by the other methods. Using the same large data set as Paez-Espino et al. (131), WiSH predicted a host at family level for 59% of the contigs (132). More recently, research studies have set up a mix of these approaches, indicating that the spectrum of the bacterial hosts of the dominant phages reflects the GM composition (127). Indeed, among 180 persistent phage clusters identified, about one third could be linked to a bacterial genus, all of them belonging to abundant taxa, such as Faecalibacterium and Bacteroides (127).

The phages may also interact directly with the host immune system and trigger immune responses. It has been hypothesized that phage tropism for the mucus could encourage the penetration of phages within the body, by mechanisms such as endocytosis and transcytosis in intestinal epithelial cells, or sampling by dendritic cells. Once the phages have been endocytosed by the dendritic cells, their nucleic acids can trigger TLR pathways, in particular TLR9-dependent process, and promote adaptive immune responses. The mechanisms of B and T-cell activation by phages are not fully clarified. However, recent findings reported that activation of B cells determines the secretion of phage-specific antibodies, both in the gut and in the systemic compartment, whereas the activation of T cells in the Peyer's patches and mesenteric lymph nodes results in production of cytokines, such as IFN-γ (130).

To date, the role of gut virome has been unknown in MDD. A recent work found, although the overall viral composition of the MDD and controls groups was not significantly different, the identification of three differential bacteriophages: *Clostridium_phage_phi8074-B1, Klebsiella_phage_vB_KpnP_ SU552A*, and *Escherichia_phage_ECBP5* assigned to *Caudovirales* between two groups. This work suggested that it is useful to investigate the roles of these phages and their bacterial hosts in the development of MDD. Interestingly, they found that some bacteriophages were also correlated with specific metabolites, hypothesizing that they may indirectly affect metabolites by targeting bacterial species (133).

Despite the limitation due to methodological issues, viral metagenomics, and database-independent whole virome analyses, *in silico* identification of novel phages as well as bioinformatics and lab-based research have contributed to clarify the “known/unknown” component of the GM and highlighted the importance of phageome to human gut homeostasis (134). Although there is still a lot of work to be done, recent efforts have been made to set up reproducible protocols for metagenomics analysis of human fecal phageomes. In this context, Shkoporov et al. (129, 135) took into account several factors known to affect data reproducibility (i.e., processing of fecal samples) and made important recommendations to achieve optimum results: rapid storage, limited freeze–thaw cycling, and spiking of fecal samples with an exogenous phage standard.

### Potentiality, Limitations and Future Directions

We describe two sides of the same coin: potentiality and limitations. Where does the needle of the scale hang?

#### Potentiality

The dramatic advances in DNA sequencing technology as well as the use of MDD animal models have afforded us a much greater understanding of the mechanisms by which GM–brain-depression communication occurs. While 16S ribosomal RNA gene sequencing has previously triumphed as the dominant identification method of microbiota species, the recent shotgun whole genome sequencing (WGS) has become much cheaper and more widely available (136). Shotgun WGS enhances detection and accuracy of GM composition and is increasingly becoming the field's new gold standard. The -omic approach with technologies including metatranscriptomics, metaproteomics, and metabolomics will consent to investigate RNAs, proteins, and metabolites in metagenome-wide association studies (MWAS). The MWAS approach shows a great potential in the identification of the GM taxonomy but also in the annotation of functions, pathways, and metabolism. At the same way, new bioinformatics software packages allow researchers to improve compositional analysis with crucial information on different functional and metabolic pathways of the GM (39).

The rapid expansion of preclinical GM–brain-depression research in the past two decades has generated realistic optimism because the GM manipulation is a relatively easy task. As reported in Bastiaanssen et al. (137), the advantages to use animal models are in maintaining the highly controlled environment and genetics, which are all factors that decrease the inter-individual variation of GM composition. In addition, a mouse has a lifespan of 2–3 years as compared to a human life expectancy of about 70 years, and this allows researchers to study the entire life cycle. GF rodents are raised in GF isolators and represent a crucial tool in defining whether the GM plays a causal role in each host function.

Apart from diet and lifestyle, prebiotics and probiotics (psychobiotics) are the first-line treatments that influence the GM architecture and thus improve the mental health with consequent positive effects also on mood (27, 137–142). They are very simple, non-invasive treatments with no significant side-effects and less expensive than antidepressant drugs. Treatment options for directly modifying the microbiota composition include also FMT (140). The potentialities linked to the positive effects of gut microbial metabolites must not be underestimated as well. Elucidating the mechanisms through which they influence the depressive behavior could develop new strategies to harness the beneficial psychotropic effects of these molecules (84). In conjunction with the GM, the oral microbiome further provides feasible merits as a diagnostic/prognostic tool as well as a therapeutic target. Moreover, the modification of oral microbiome simply by improvement of dental hygiene and/or supplementation with probiotics can modulate the pathogenesis of disease (104). Consequently, oral- and intestinal-specific bacterial species and their products may be potential biomarkers for the prevention and clinical diagnosis of MDD.

The potentiality of phages lies in their use not only as individual modulators of the GM in a variety of infectious and non-communicable human diseases including MDD but also as potential future treatments against these pathologies along with antibiotic-resistant bacterial infections. Moreover, different studies are investigating the role of phages in the dysbiosis that accompanies various pathological conditions. Thanks to recent progresses in the detection of phage–bacteria pairs, it is needed to perform longitudinal studies to identify possible relationships between temporal shifts in bacteria and their associated phages and to explain whether phages may provide a contribution to dysbiosis and disease or, on the contrary, help to maintain microbiota stability by preserving bacterial diversity.

#### Limitations

A major challenge of these studies lies in translating the animal model in human clinical settings. First, a healthy GM has not been still defined, and the term dysbiosis (altered or unbalanced GM) is a vague concept. GM composition varies widely between individuals, and it is strongly influenced by age, because of confounding factors such as illness, diet, medications, and stress. Thus, a large-scale population is needed to characterize an optimal GM configuration. In this context, the American Gut and British Gut Microbiome projects are joint crowd-funded initiatives where volunteers donate their biological samples with the aim to collect information and give a better idea of what constitutes a normal GM. Additionally, making arrangements on methods related to standardizing collection, storage, and processing, or clearly communicating how procedures vary, is a crucial point to global depression research (39). This includes creating at-home collection methods that are simple and robust. A recent proof of concept study has demonstrated that the analyses performed in soiled toilet tissue stored at room temperature for 7 days can be comparable to use an immediately frozen fecal sample (37). Indeed, different storage systems that allow for longer fecal sample stability are proving comparable to the immediate freezing without preservation gold standard. However, while collection and storage of fecal samples is becoming increasingly simplified, further effort should be focused on other types of variability associated with collection that are often ignored in GM analysis: various forms of bowel preparation that may take days to make a full recovery, temporal stability, medication use, eating behaviors. If fecal GM samples can be individually stable over many months, a single course of antibiotic treatment may change microbial diversity for over a year. Moreover, as MDD is often accompanied by circadian disruption and shifts in eating habits, it is further significant to make attention to these variables at time of collection, which is critical for future research. The International Human Microbiome Standards project has been set up with the aim of developing standard operating procedures designed to improve data quality and comparability in the human microbiome field (39).

Although there is a great enthusiasm on the efficacy of psychobiotics on MDD through GM manipulation, further investigations are warranted. Longitudinal studies with pro-prebiotics and other microbiota targeted interventions are required to validate this approach. It is over 100 years ago since George Porter Philips put forward the concept of treating melancholia with lactobacillus; with more clinical research, we may be able to validate how much he was ahead of his time.

Modern high throughput sequencing technologies have played a significant role in improving our understanding of the human gut phageome. However, much of the generated sequencing data remains uncharacterized. Even those phages that are successfully characterized only provide limited insight into their associated biological properties, and thus, most viral sequences have been cataloged as “viral dark matter” (143). Moreover, little progress has been made in the isolation and characterization of novel gut phage-host pairs. In recent years, efforts have been performed to overcome bottlenecks. This requires the development of database-independent bioinformatics pipelines, universal and easily reproducible methods that reduce bias during viral enrichment, nucleic acid extraction, and sequencing library preparation. *In silico* analyses need to be performed using database-independent methods that permit a complete virome analysis using benchmarked criteria. The development of a sequence-based taxonomic scheme is necessary to accelerate the rapid expansion of phage sequences because of high-throughput sequencing technology. The current contradictions among different gut virome studies need to be rectified and clarified. Recent findings have provided significant understandings, but they also emphasize how little we know about this important and enigmatic component of our gut.

Also, the interesting clinical work performed in MDD and phageome remain preliminary (133); the influence of environmental and demographic factors cannot be completely excluded.

Concerning the oral microbiome, the studies available are based on relatively small samples and cross-sectional surveys. Moreover, they are not designed to consider the progressive nature of oral dysbiosis and mental disorders over time. The absence of consensus in the definition of the oral dysbiosis, the methodological variants in the collection of the bacterial samples and the targeted species generate further heterogeneity in the results. In addition, the evidence for a two-way causal link between the oral microbiota and mental disorders is limited in humans due to a lack of value confounding factors. If age and gender are sometimes considered, important parameters, such as lifestyle habits (e.g., smoking, diet, oral hygiene) or functional or immune disorders related to mental disorders or somatic pathologies, are rarely considered in the studies (118).

#### Gaps to Be Bridged

An area that remains poorly explored is the study on enteroendocrine cells that communicate with and activate the vagus nerve. To know how these cells operate will allow researchers to develop not only more effective pro-prebiotics formulations but also to explore whether pre-probiotics could stimulate vagal afferents so to reinforce the antidepressive effects (39).

Another field to continue developing is the FMT, which could become useful in the future as a therapy for MDD (137).

The sex-dependent differences in GM composition are another important gap requiring major attention: it is well-known that females are most likely to suffer depression than males. This sexually dimorphic GM has been termed the “microgenderome” and it is crucial to deep the investigations on this topic (144).

We are just beginning to understand what role the GM plays during brain development. Early life is a period of rapid shifts, and the developing brain is susceptible to most internal and external adverse influences. In addition, many metal disorders can derive from early exposure to stress and inflammation. Therefore, improving our knowledge of the GM and oral microbiota impact at this stage will also have a significant influence on diagnosis and possible treatments.

## Conclusions

**Figure 2:**
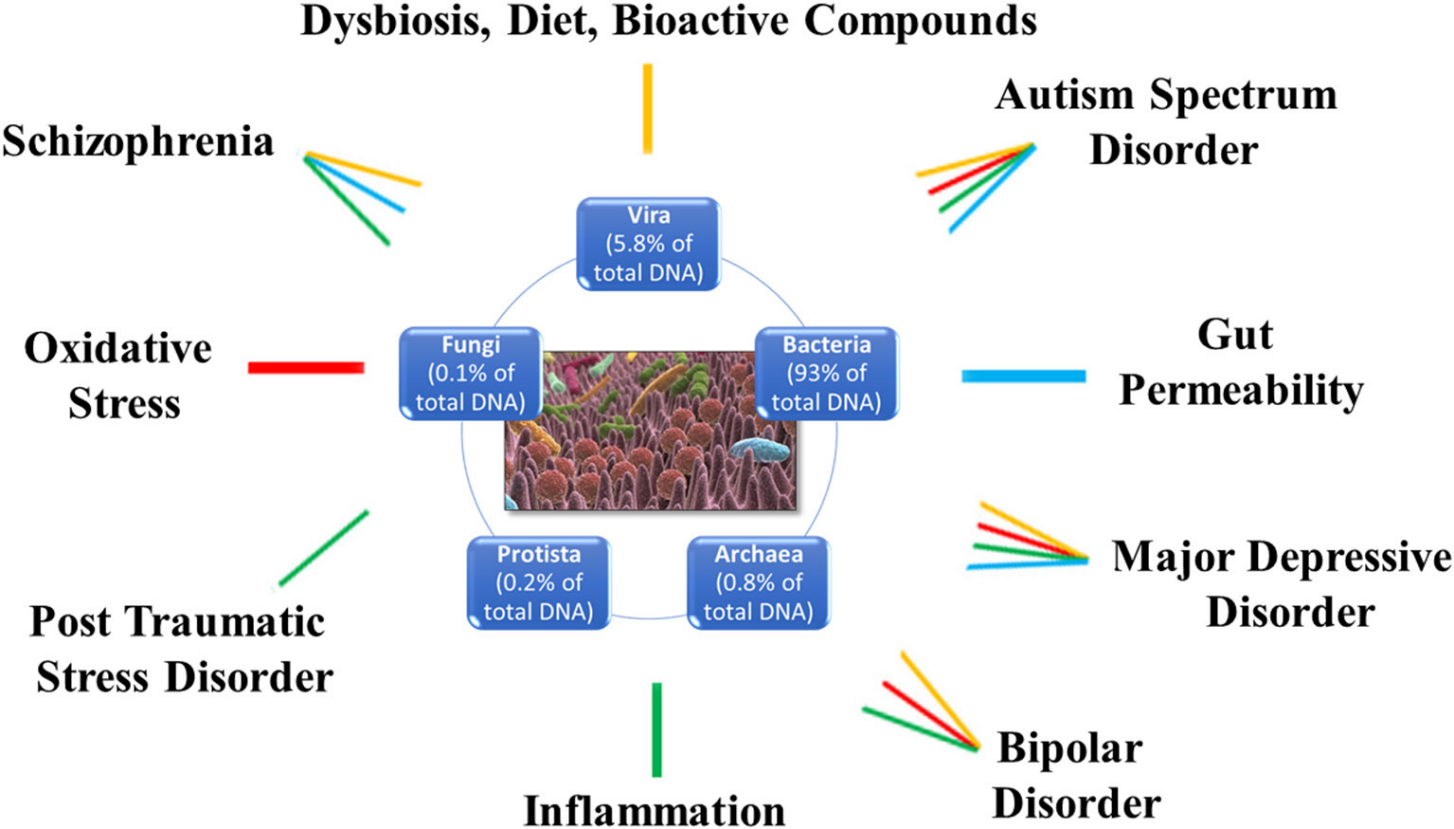
Schematic representation of the different molecular pathways linking the microbiota with different psychiatric diseases including major depressive disorder. Each pathology is associated to specific altered biological processes: major depressive disorder and autism spectrum disorder are associated to dysbiosis, and alterations in diet and bioactive compounds (yellow), oxidative stress (red), inflammation (green), and gut permeability (light blue); schizophrenia is associated to dysbiosis, and alterations in diet and bioactive compounds (yellow), inflammation (green), and gut permeability (light blue); bipolar disorder is associated to dysbiosis, and alterations in diet and bioactive compounds (yellow), inflammation (green), and oxidative stress (red); post-traumatic stress disorder is associated to inflammation (green).

The study of the microbiome (gut-oral)-brain axis is revolutionizing our understanding of the mechanisms underlying MDD. Based on the availability of studies and research, we tried to rebuild the complex routes of communication between GM and brain-depression. The result is that the role of GM in MDD lies at the intersection of endocrine, immune, neural systems, oxidative stress, microbial neurotrasmitters, and metabolites. On the other hand, the unbalance of the cross-talk between gut and brain is shared also by other psychiatric disorders such as autism spectrum disorder, bipolar disorder, posttraumatic stress disorder and schizophrenia (Figure 2), suggesting a possible common mechanism of action. Another important and emerging actor is the oral microbiome and its connections with both the gut and the brain that, both directly and indirectly, might impact on the GM alterations as well as the neuroinflammatory processes involved in MDD (Figure 1).

The identification of biomarkers associated with diagnosis and treatment is crucial since no definitive biomarkers are available for MDD. It is thus certainly plausible that an individual's microbiome (gut/oral) fingerprint could be a further component of a MDD biomarker panel and, consequently, could indicate whether microbiome (gut/oral)-based treatment may be a useful component of the therapeutic collection for MDD treatment.

## Author Contributions

CS, MM, and NC managed the literature searches and contributed to the first draft of the manuscript. CS, MM, NC, EM, and AC contributed to the revision. AC revised all the versions of the manuscript and approved the final one. All authors have contributed to and have approved the final manuscript.

## Funding

CS, MM, NC, and AC received funding from Italian Ministry of Health (Ricerca Corrente). AC was also supported by the PSR (Piano Sostegno Ricerca) from the University of Milan.

## Conflict of Interest

The authors declare that the research was conducted in the absence of any commercial or financial relationships that could be construed as a potential conflict of interest.

## Publisher's Note

All claims expressed in this article are solely those of the authors and do not necessarily represent those of their affiliated organizations, or those of the publisher, the editors and the reviewers. Any product that may be evaluated in this article, or claim that may be made by its manufacturer, is not guaranteed or endorsed by the publisher.
